# Effect of Local Heat Pipe Cooling on Throughput Distribution and Thermal Homogeneity in a Binary Melt Pre-Distributor for Polyolefin Extrusion

**DOI:** 10.3390/polym14112271

**Published:** 2022-06-02

**Authors:** Christian Hopmann, Lisa Leuchtenberger, Malte Schön, Lena Wallhorn

**Affiliations:** Institute for Plastics Processing (IKV) in Industry and Craft at RWTH Aachen University, Seffenter Weg 201, 52074 Aachen, Germany; sekretariat@ikv.rwth-aachen.de (C.H.); malte.schoen@ikv.rwth-aachen.de (M.S.); lena.wallhorn@rwth-aachen.de (L.W.)

**Keywords:** thermal homogenisation, pre-distribution, heat pipe, blown film extrusion, CFD

## Abstract

In polymer blown film extrusion, inhomogeneous die temperature distributions lead to an inhomogeneous temperature and cause film thickness variations. To avoid an inhomogeneous film thickness and to achieve good film qualities, thermal homogenisation of the melt is necessary. Therefore, a new approach for cooling hot spots with heat pipes is investigated. CFD Simulations in OpenFOAM show that heat pipes can be used to influence melt temperatures locally in the places in which a temperature reduction is required. Since the outlets interact in a pre-distribution die, one heat pipe is not sufficient to homogenise the temperature at every outlet to similar temperatures. Two heat pipes show much better results with lower average temperature deviations between the distributor outlets. In order to equalise the temperature at all outlets, at least one heat pipe per outlet will be required.

## 1. Introduction

**Figure 1:**
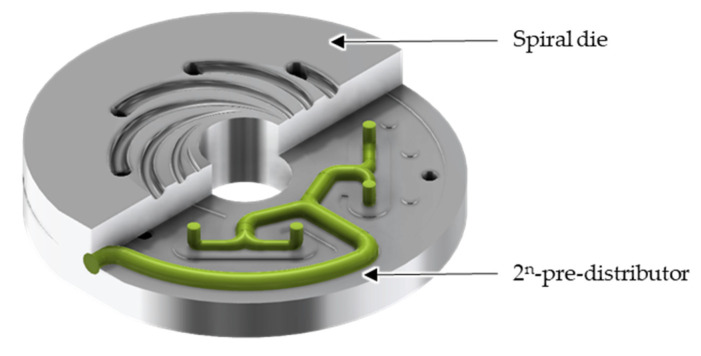
The 2^n^-pre-distributor in combination with a spiral die as main distributor in a blown film process.

About 39% of all plastics are processed into packaging [1]. In turn, 49% of all packaging is produced using extrusion processes, such as blown film extrusion [2]. In blown film extrusion, plastics granules are melted and homogenised in an extruder. Afterwards, the melt is transformed into a tubular cross-section in an extrusion die consisting of a pre-distributor and a main distributor, normally a spiral die (Figure 1). At the die outlet, the melt is hauled-off and inflated. Air cooling then initiates the cooling process via convection. At the same time, the melt is stretched in the circumferential direction by the blow up process and in the extrusion direction by the haul-off. A great advantage of this process is the variable film width, which can be easily adjusted by the blow-up ratio without changing the die. Moreover, the induced biaxial stretching renders a stronger and less permeable film [3,4].

The most important quality aspects of films are the homogeneous film thickness and film flatness. Packaging films, in particular, often consist of multilayer films, where each layer performs a specific function. If one of the layers contains thin spots, functions such as keeping oxygen or water out can no longer be provided, which can lead to, e.g., spoiled food [4]. To ensure the reliable function of every layer, the respective thinnest spot has to reach a specified minimum thickness, which leads to unnecessary material usage in case of inhomogeneous film thickness distribution. Whether the film has a homogeneous layer thickness depends on various influences. One influence is the throughput distribution at the die outlet [5]. Circumferential segments of the die outlet that carry more of the melt cause an increased wall thickness in the film. Another influence is the temperature and the viscosity distribution at the die outlet [6]. Areas with higher melt temperatures are more stretched in the blow-up-process due to the lower extensional viscosity. Therefore, these areas appear as thin sections in the film.

To reach a perfectly homogeneous blown film, a large number of empirical and simulative studies from research and industry has been carried out. They find a temperature influence on the melt distribution and temperature distribution in extrusion dies. Astarita et al., pointed out, using the example of the mandrel holder die, that non-uniform dissipation and asymmetric heat conduction in the die material can lead to an inhomogeneous temperature distribution in the die [6]. The fact that inhomogeneous temperature distributions at the die outlet can influence the throughput distribution was shown by Vergnes et al., using the example of a flat die [7]. This also includes the influence of shear heating in the extrusion die [8,9,10,11]. Zatloukal et al., performed a non-isothermal flow analysis on the flow channel in a spiral die [12]. In their work, the influence of the temperature distribution calculations was investigated. A comparison of the non-isothermal and isothermal calculations show that the simulation results of the non-isothermal calculations agree better with experimental data. This observation explained the viscous dissipation causing a temperature increase and viscosity decrease. Another study on spiral distributor dies was carried out by Skabrahova et al. [13]. They simulated the flow channel of an axial spiral distributor to investigate whether the assumption of a homogeneous throughput and temperature distribution at the inlet to the spiral distributor is permissible. By simulating different asymmetric inlet conditions at the spiral distributor inlets, they showed that some equalisation processes for thermal and rheological homogenisation take place along the spirals. Nevertheless, inhomogeneous inlet conditions lead to a significant deterioration of the melt distribution profile at the die exit. However, these considerations on non-uniform inlet conditions are not based on real measurements, since only different hypothetical scenarios were run [13]. The general importance of thermal influences on the throughput distribution in extrusion dies is also emphasized by Pittman [14]. In a review of the state-of-the-art extrusion die simulation, he explains that both dissipative shear heating and temperature distribution in the die can negatively affect the velocity distribution at the die exit. Pittman recommends introducing differences between the melt and flow channel wall temperatures in a targeted manner in order to influence and equalize the flow in this way. Burmann et al., also discussed the temperature influence in spiral mandrels. They give general advice on how spiral mandrel dies can be optimized with regard to processing temperatures [15,16]. The installation of internal temperature control systems (water or oil temperature control), thermal separation by insulating layers or improvement of heat exchange (e.g., by ceramic fins) are recommended as possible solutions to improve thermal conditions. In later contributions, it was pointed out that the plastic melt is subjected to locally different dwell time and temperature-related stresses in the overall extrusion die system, depending on the design of the main and pre-distributor [17]. Saul investigated the automated die design of radial spiral dies in his dissertation [18]. He suggested for future investigations to consider non-isothermal calculations, due to local melt shear heating. Moreover, he pointed out the expansion potential of his investigations, which optimise the temperature distribution in the die. The temperature effect in a pre-distributor was analysed four years later by te Heesen, using the example of a centrally fed star pre-distributor [19]. The non-isothermal flow simulations, taking into account both shear heating of the melt and heat conduction in the die material, show that the shear stresses and the pressure drop are influenced by the dissipative shear heating. However, no influence on the throughput distribution was observed, which can be attributed to the rotationally symmetric geometry of a star distributor. Furthermore, te Heesen notes that the temperature influence can affect the throughput distribution in more complex pre-distributors, such as a 2^n^-distributor. The analysation and homogenisation of the thermal effects in a 2^3^-pre-distributor was carried out by Yesildag [5,20]. The different melt temperatures cause viscosity differences between the outlets, which lead to an inhomogeneous mass throughput. Yesildag’s approach to homogenising the throughput distribution was to integrate heating cartridges and thermally conductive inserts to homogenise the temperature distribution in the die. Prior to practical trials, the effect of heating cartridges and thermally conductive inserts was investigated using CFD Simulations. He succeeded in homogenising the melt distribution at the die outlets, but to optimise the temperature distribution for one process point, 12 heating cartridges are necessary. Each of them has a different temperature, which makes this approach highly process point dependent and requires a separate control circuit for each cartridge. Another disadvantage is the occurrence of overheated spots in the die, which rules out the usage of thermally sensitive materials such as EVOH or PVDC. In addition, heating cartridges require high maintenance due to their short service life. Therefore, a simpler approach in the form of adjustable nozzles was developed [21]. Through adjustable nozzles at every pre-distributor outlet, an equal distribution of the melt flows can be achieved. However, the temperature distribution remained outlet dependent. Therefore, film thickness variations will appear, despite a melt distribution homogenisation in the pre-distributor.

In addition to the described direct heating methods for extrusion dies, which are based on electricity, another method is indirect heating by liquids. Indirect heating is preferred in the processing of elastomers [22]. The key feature that distinguishes heating with liquids from electrical heating is the ability to provide heat regardless of ambient conditions. This proves to be an advantage in the typical, relatively low temperatures that are common in the processing of elastomers [22]. Fluid heating is also occasionally used in relatively small dies for thermoplastics. To add or remove heat from a specific area, such as from the exit zone of the mandrel of blown film dies, oil heating is used. The reassembly of the dies, when they are changed or serviced, is usually more complicated than with direct electric heating, because of the heating flow channel cleaning [22]. A further disadvantage of liquid heating is the technical complexity and the cost in cases where different die zones must be maintained at different temperatures. Current technology requires a separate heating unit for each temperature zone.

Since the temperature has a major influence on polymer viscosity, it affects not only the flow resistances and thus the mass flow distribution, but also the extensional viscosity beyond the outlet of the main distributor. During inflation, the melt parts of lower extensional viscosity can be stretched more easily, resulting in thin spots in the film. To avoid thickness variations in the film, this paper’s approach is to develop an alternative thermal die control with heat pipes in order to avoid producing temperature hot spots in the die, which is not only less process point dependent, but also requires low maintenance and can be easily retrofitted.

**Figure 2:**
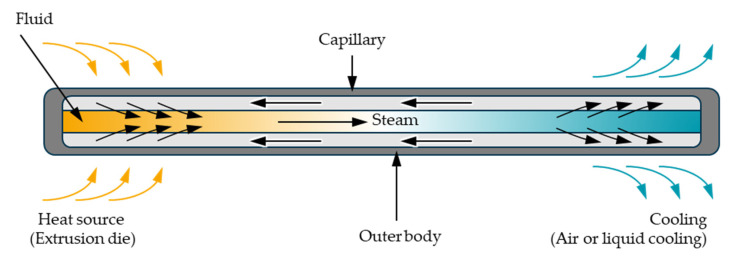
Schematic of a heat pipe.

Heat pipes are already used in many areas such as electronics cooling and aerospace [23,24,25]. They consist of a closed outer body, usually cylindrical in shape, with a capillary structure on the inner walls and are partially filled with a fluid [25,26]. This structure is shown in Figure 2.

When the temperature at one end of a heat pipe is increased, the fluid starts to evaporate causing localised cooling. A pressure gradient is established inside the cylinder, and the gas flows to the colder end. Here the evaporated fluid cools down and condenses. Through the capillary structures, the fluid is transported back to the warmer end. The process repeats as long as there exists a temperature gradient between the heat pipe’s ends. Due to the high enthalpies of evaporation and condensation, heat pipes are able to transport large amounts of heat. If, for example, a solid copper rod with a diameter of 8 mm and a length of 300 mm is used to transfer a heat quantity of 100 W, a driving temperature gradient of theoretically 1493 °C would be required. According to heat pipe manufacturers, a heat pipe achieves the same heat transfer with a driving temperature gradient of approx. 0.5 K [24]. Through the choice of cylinder material and working fluid, it is possible to create heat pipes for nearly every application temperature range. According to Stephan, heat pipes with a copper cylinder and water as working fluid are suitable for most polymer extrusion application temperature ranges [25]. The heat pipe performance can be calculated with Equation (1) and is dependent on the temperature difference between the heat pipe ends, the chosen material combination, the cross section and the capillary structure [26].

(1) $$\Delta T\;=\;\overset{\cdot }{Q}R_{\mathrm{tot}},$$

**Figure 3:**
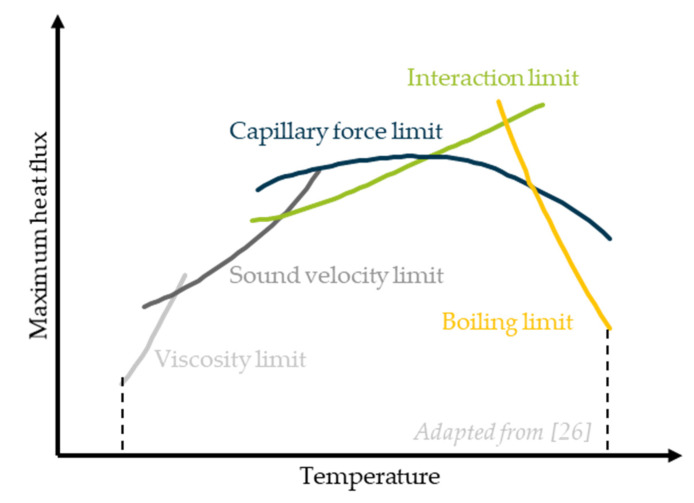
Performance restrictions of heat pipes [26].

R_tot_ is the total thermal resistance, including the radial resistance between external heat source and evaporator wall and the axial resistance of the steam flow. The heat pipe performance or heat flux density is restricted by several physical boundaries shown in Figure 3 [26]:

- Viscosity limit
  This limits the heat flux density at working temperatures just above the fluid’s melting point. Here, the pressure difference driving the steam between evaporator and condenser is small, and the steam flow is determined by high viscous forces and can even be interrupted.
- Sound velocity limit
  If the vapour reaches the sonic velocity at a certain evaporator temperature, this velocity cannot be exceeded even by reducing the steam pressure in the condenser. This limits the maximum heat transfer.
- Interaction limit
  At high heat flux density, liquid is entrained by the vapour, and a partial drying out of the capillary leads to a breakdown in the liquid flow.
- Capillary force limit
  The capillary force limit is reached when the flow losses of the liquid heat transfer medium are greater than the existing capillary pressure. This scenario is dependent on the temperature difference between evaporator and condenser.
- Boiling limit
  Bubbles boiling in the capillary restrict the flow of the liquid or stop it.

If one of these five limits is exceeded, the capillary structure in the heating zone dries out. This significantly increases the thermal resistance in the evaporator. Since this thermal resistance has a significant influence on the overall thermal resistance, this results in a significant temperature increase in the heat source.

Heat pipes have short reaction times, a compact design and enable operation without an external power source [27,28]. Lakemeyer et al., already investigated the use of heat pipes or the heat pipe principle for temperature control of extruder screws [29]. In this case, the extruder screw acts as a heat pipe, the temperature peaks in the screw are reduced and the thermal homogenisation of the screw takes place through heat equalisation processes. Furthermore, Kartelmeyer et al., used heat pipes to control the temperature of injection moulds, as this approach is a low-maintenance and efficient way of transporting heat [27,28].

To compare different heat pipes, a specific performance rating can be useful. One possibility is to calculate the merit number M, which is composed of the surface tension σ, the evaporation enthalpy $\Delta h_{e}$ and the kinematic viscosity $\nu_{l}$ (Equation (2)).

(2) $$M=\frac{\sigma \Delta h_{e}}{\nu_{l}},$$

The performance number M should be as large as possible in the application-specific temperature range.

Considering the high performance of heat pipes, in this paper it is investigated whether a maximum of two heat pipes per pre-distribution half is sufficient to achieve thermal homogenisation. For this purpose, CFD simulations are performed to determine suitable heat pipe positions. Starting from an initial baseline simulation without heat pipes, four suitable positions are defined. To assess the suitability of the heat pipes, the average temperature deviations in each outlet, the thermal melt homogeneity and the melt distribution are considered. Two promising positions are further investigated by varying the heat pipe power up to 80 W for one heat pipe or 210 W in the case of two heat pipes. The effects of heat pipes with different cooling capacities need to be investigated to ensure that an appropriate heat pipe capacity is considered when a heat pipe is specified for a practical application. The aim is to achieve temperature and melt distribution deviations of less than 1% between the outlets.

## 2. Materials and Methods

In order to investigate local cooling through the operating behaviour of the heat pipes and their effect on melt temperature and melt distribution, CFD simulations were performed using OpenFOAM software (OpenFOAM Foundation Ltd., London, UK). In Section 2, the authors present the material and methods used for the CFD-Simulations.

### 2.1. Materials

As low-density Polyethylen (PE-LD) is the most processed material in the blown film extrusion, a PE-LD type 2101N0W from SABIC, Riyadh, Saudi Arabia is investigated in the simulations. In order to describe the shear-thinning, temperature-dependent material behaviour, the combined approach of Carreau and Williams, Landel and Ferry (WLF) is suitable [18,19,21,26]. The Carreau and WLF parameters were determined from high-pressure capillary measurements at IKV. The shear viscosity η in dependence of the shear rate $\overset{\cdot }{\gamma }$ and temperature T is described in Equations (3) and (4).

(3) $$H(\overset{\cdot }{\gamma })=\frac{18,757.01}{{\left(0.86\;\overset{\cdot }{\gamma }\right)}^{0.703}},$$

(4) $${\mathrm{lg}(a}_{T})=-\frac{8.86\left(T\;-\;257.481\;K\right)}{101.6\;K\;+\;T\;-\;257.481\;K\;},$$

Another relevant material parameter is the specific melt density. The pressure and temperature dependent density was determined using p-v-T measurements. As the density is almost constant at 200 bar and temperatures between 180 °C and 230 °C, incompressibility of the melt is assumed. For the simulations, an average density value of 769 kg/m^3^ is set. To perform a non-isothermal simulation, thermal properties are required as well. The thermal conductivity of 0.282 W/(Km) at 473.15 K was determined in a laser flash analysis (LFA). The specific heat capacity of 2.722 J/(gK) at 473.15 K was determined by differential scanning calorimetry (DSC).

### 2.2. Start and Boundary Conditions

**Figure 4:**
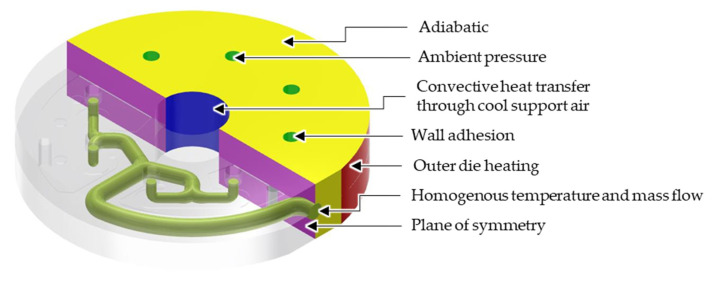
Start and boundary conditions.

In accordance with common practice, a steady state with laminar, perfectly wall-adherent and incompressible flow is considered. Moreover, a homogeneous temperature and velocity at the die inlet are assumed (Figure 4) [5,20,21].

### 2.3. Meshing

The die geometry analysed is based on the work of Yesildag [5]. In the pre-processing, all chamfers, holes and threads are neglected. Furthermore, the die geometry is reduced to one half of the symmetrical die. Since a future comparison of heat pipes and fluid oil heating is intended, an easy mesh adjustment is advantageous. Therefore, the use of the immersed boundary method is appropriate at this point [30]. The defining feature of this approach is the possibility to perform the entire simulation on a fixed Cartesian grid.

**Figure 5:**
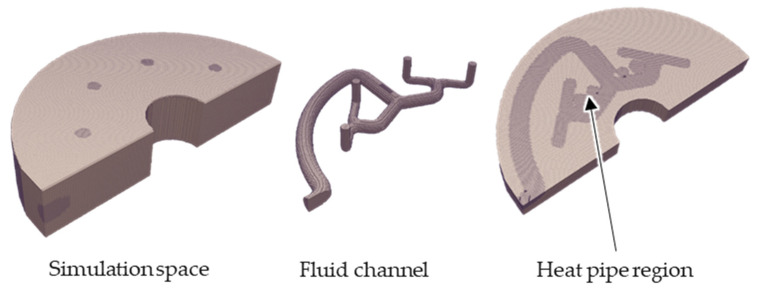
Meshed simulation space and flow channel geometry.

To discretise the pre-distribution geometry, the mesh generator snappyHexMesh is used, which performs a representation of the complete computational domain by hexahedron-shaped control volumes. All elements are first assigned a very high-flow resistance. For this purpose, according to Khadra et al., at the moment the conservation equation is extended with a term in which a proportionality factor for the velocity vector is integrated. The proportionality factor represents the inverse Darcy coefficient, i.e., the permeability of the material [30]. If this value is very high, the flow of the fluid through this geometry volume is suppressed, representing the steel body. The flow channels are then taken into account by means of an adjustment of the local flow resistances. In this process, the flow resistance is reduced in all elements of the die grid that are located within the flow channel geometry. Although a coarser partitioning is sufficient outside the flow channel, since only the heat conduction is calculated, the mesh in the flow channel is refined to represent flow effects sufficiently accurately. Mesh independence checks have shown that a one-level refinement is sufficient. Furthermore, areas where an integration of heat pipes would potentially be useful are refined. For reasons of symmetry, it is sufficient to perform the calculations for only one half of the mould. This results in a mesh with 620,141 volumes, which is shown in Figure 5 and leads to a computation time of about 3.5 h.

### 2.4. Solver

A solver based on the SIMPLEC (Semi-Implicit Method for Pressure Linked Equations-Consistent) method is used [31,32]. The solver is implemented in the open-source finite volume software OpenFOAM (OpenFOAM Foundation, London, UK). The differential equations of mass, momentum and energy conservation are solved for the variables pressure, flow velocity and temperature, whereat the shear heating is additionally taken into account for the latter. This flow solver was extended in [33] by an immersed boundary approach according to Khadra et al. [30].

∇ · **u** = 0, (5)

∇**p_norm_** = ∇ · (**u** × **u**) − ∇ · **τ_norm_** − δ**u**, (6)

(7) $$0=\nabla \cdot \left(\mathrm{uT}\right)+\nabla \cdot \left(\alpha \nabla T\right)+\frac{\tau_{\mathrm{norm}}}{c_{p}}:(\nabla \;\times \;u),$$

In the conservation equations, **u** is the vector of velocities and **p**_**norm**_ is the pressure normalized to the density ρ. Moreover, **T** is the temperature and α is the local thermal diffusivity. The zero-velocity Dirichlet condition is used as a penalty condition. The inverse Darcy coefficient δ in the momentum equation is 0 for the fluid region and becomes >>0 for the die region, which corresponds to a complete suppression of flow. In [33], the temperature distribution could not yet be described. Therefore, a heat equation, including the shear heating calculated from the shear stresses in the energy equation, is implemented as follows [34].

(8) $$0=\nabla \cdot \left(\mathrm{uT}\right)+\nabla \cdot \left(\alpha \nabla T\right)+\frac{\tau_{\mathrm{norm}}}{c_{p}}:(\nabla \;\times \;u),$$

(9) $$\tau_{\mathrm{Norm}}=\nu (\left(\nabla \;\times \;u\right)+(\nabla {\;\times \;u)}^{T}),$$

**τ_norm_** is the tensor of the shear stress normalized to the density ρ, c_p_ is the local specific heat capacity and **ν** is the kinematic viscosity (nu).

To take into account the highly variable placement of the heat pipes a similar approach is applied. For simplicity, the heat pipes are considered as simple cylindric geometries, functioning as a constant heat sink. In those cells defined as part of the heat pipes, a constant cooling rate in K/s is assumed. The cooling rate is freely adjustable. Therefore, the energy equation is extended as follows:

(10) $$0=\nabla \cdot \left(\mathrm{uT}\right)+\nabla \cdot \left(\alpha \nabla T\right)+\frac{\tau_{\mathrm{norm}}}{c_{p}}:\left(\nabla \times u\right)+\varepsilon C,$$

For die regions without heat pipes ε is 0. Cells overlapping with the heat pipe geometry (ε = 1) act as a heat sink for C < 0 and as heat source for C > 0. The immersed boundary method is reasonable at this point, laminar material behaviour is assumed and no turbulent wall conditions are required. In addition, the heat fluxes at the boundary between solid steel and liquid melt are expected to be low so that the assumption of perfect heat transfer between solid and liquid is reasonable.

## 3. Simulation Results and Discussion

**Figure 6:**
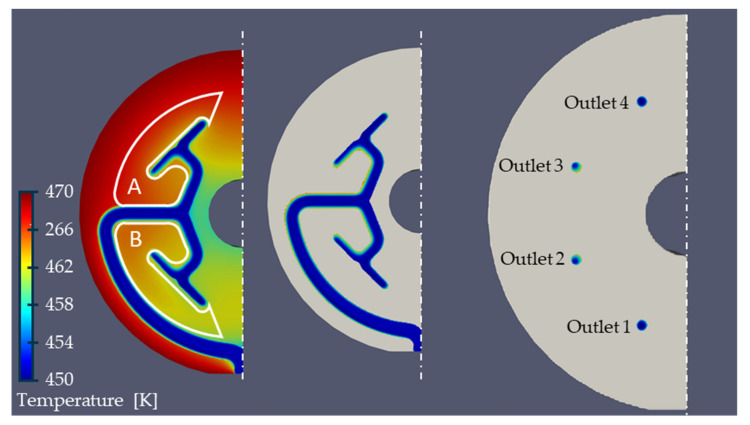
Temperature distribution in a 2^n^-pre-distributor without heat pipes.

In order to determine the temperature distribution without thermal homogenisation, a first basic simulation is performed without heat pipes. According to Yesildag, the temperature and throughput variation between the outlets increases as the difference between the melt and die temperature increases. Moreover, the shear heating effect is affected by the total mass throughput. A homogeneous inlet mass flow of 100 kg/h with a constant melt temperature of 453.15 K and an outer die temperature of 473.15 K is assumed, which is in the range of realistic processing temperatures [35]. The temperature of the cool air in the centre of the die is assumed 293.15 K; this causes a heat transfer coefficient of 18 W/(m^2^*K) [5]. Since the top and bottom of the die are in contact with other heated components of the extrusion line, e.g., the spiral die, it can be assumed that the heat loss is comparatively low. Therefore, the top and bottom are considered adiabatic. Figure 6 shows the resulting temperature distribution both in the die and at the outlets. The outlets are numbered from 1 to 4. It becomes clear that the temperature in the outlet is not homogeneous. In addition, the temperature at outlets 1 and 4 seems to be lower than at outlets 2 and 3, which is in good agreement with the work of Yesildag and Hopmann and shows that the immersed boundary approach works [5,20,21].

**Figure 7:**
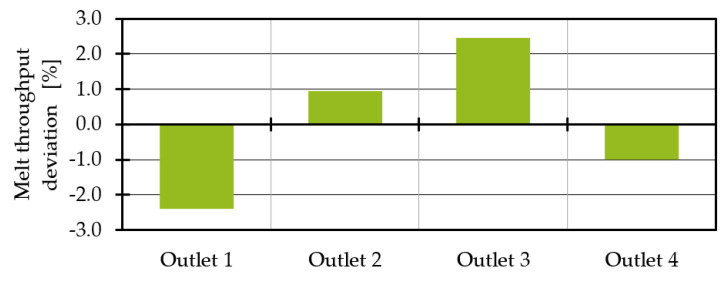
Melt throughput deviation without heat pipes.

**Figure 8:**
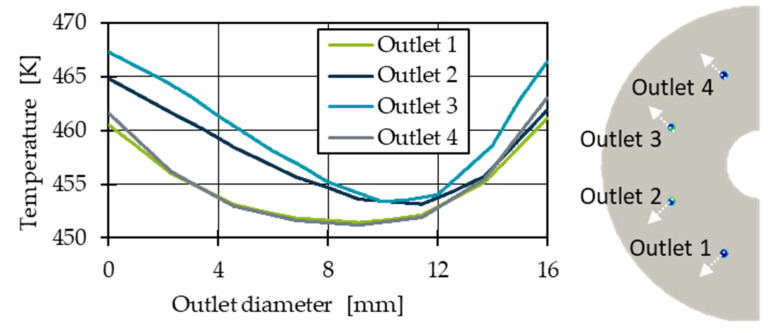
Temperature distribution in the cross section of each individual outlet.

A consideration of the mass flow distribution in dependence of the outlets (Figure 7) as well as the representation of the temperature across the outlet diameter shown in Figure 8 supports this thesis. Due to a shielding effect of the flow channel, the die region A is warmer than the die region B (Figure 6), which leads to a slightly warmer melt at outlet 3 where a temperature maximum of 460.63 K on average is present. Outlet 2 shows a temperature of 459.63 K. The minimum temperature of 456.32 K is found at outlet 1. The temperature differences between the minimum and maximum temperature of 4.31 K meaningfully impacts the shear and extensional viscosity. The shear viscosity drops by 8.9% (Equation (4)).

**Figure 9:**
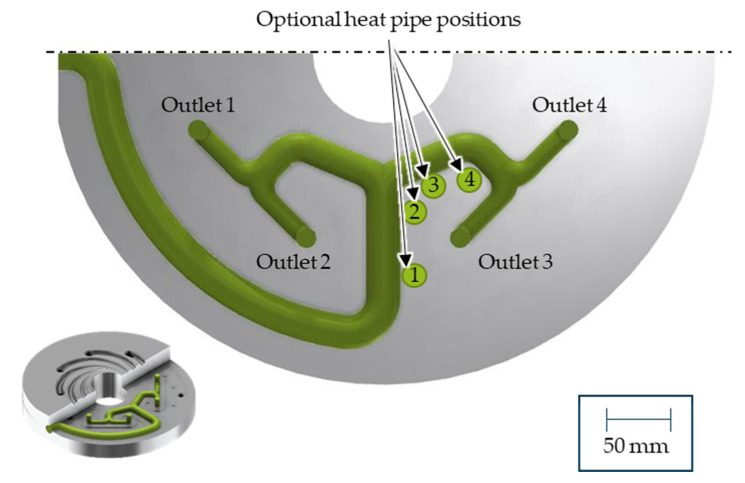
Heat pipe position 1–4.

**Figure 10:**
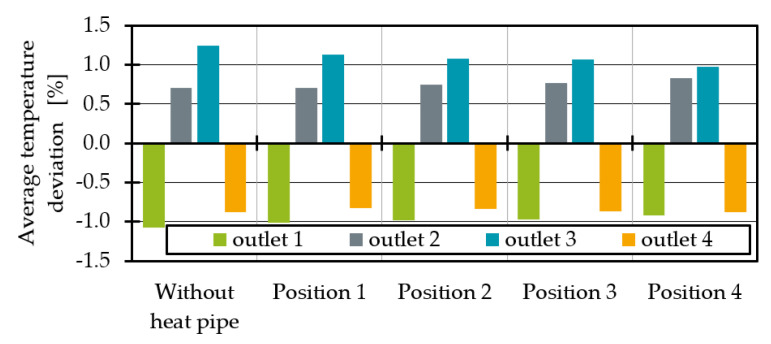
Average temperature deviation for different heat pipe positions.

To homogenise the temperature and the mass throughput, the third outlet was cooled. Therefore, four different heat pipe positions are investigated, which are shown in Figure 9. In each simulation, one heat pipe with a radius of 3 mm and a length of 30 mm was integrated. Since the pre-distributor is an interacting system where the effect on one outlet leads to deviations at all other outlets, the efficiency of the heat pipes and the average of the temperature deviation across all four outlets (Equation (11)) were compared. The resulting average temperature deviations for a cooling rate of −25 K/s, which corresponds to a heat transfer capacity of 44 W, are shown in Figure 10. According to a manufacturer’s data sheet, a heat pipe with a diameter of 6 mm and a length of 100 mm can reach up to 137 W [36].

(11) $$\Delta T_{\mathrm{average},\;x}=\frac{\frac{\sum_{j=1}^{n_{x}}T_{j}}{n_{x}}-\frac{\sum_{x=1}^{4}\sum_{j=1}^{n_{x}}T_{j}}{\sum_{x=1}^{4}n_{x}}}{\frac{\sum_{x=1}^{4}\sum_{j=1}^{n_{x}}T_{j}}{\sum_{x=1}^{4}n_{x}}},$$

The average temperatures at the third outlet are decreasing the closer the heat pipe is positioned to the outlet. This is caused by the large surrounding melt-carrying surface. Position 4 is nearly surrounded by melt flow channel walls, which is resulting in a larger heat flow at constant heat flow density, and thus a smaller temperature difference between outlet 1 (minimum temperature) and outlet 3 (maximum temperature). Without heat pipes the temperature difference is 4.31 K. With one heat pipe at position 4 with a cooling rate of −25 K/s it decreases to 3.51 K.

As the temperature distribution is not homogeneous over the outlet surface (Figure 6 and Figure 8), e_thermal_ was used to evaluate the thermal homogenisation at all outlet’s cross sections. The indicator e_thermal_ is often used to evaluate mixing devices and allows a comparison of the temperature distribution in one cross section compared to the temperature distribution in another cross section [37,38]. The calculation of e_thermal_ is given in Equations (12) and (13), where σ describes the thermal homogeneity at the outlet without heat pipe influence and the outlet with heat pipe influence with A = total area, A_i_ = cell area, υ_i_ = cell temperature, $\overset{-}{\upsilon }$ = average temperature in the outlet plane.

(12) $$e_{\mathrm{thermal}}=\frac{\sigma_{\mathrm{without}\_\mathrm{heat}\_\mathrm{pipe}}-{\;\sigma }_{\mathrm{with}\_\mathrm{heat}\_\mathrm{pipe}}}{\sigma_{\mathrm{without}\_\mathrm{heat}\_\mathrm{pipe}}},$$

(13) $$\sigma =\sum_{i=1}^{n}\frac{A_{i}{\left(\upsilon_{i}-\overset{-}{\upsilon }\right)}^{2}}{A},$$

**Figure 11:**
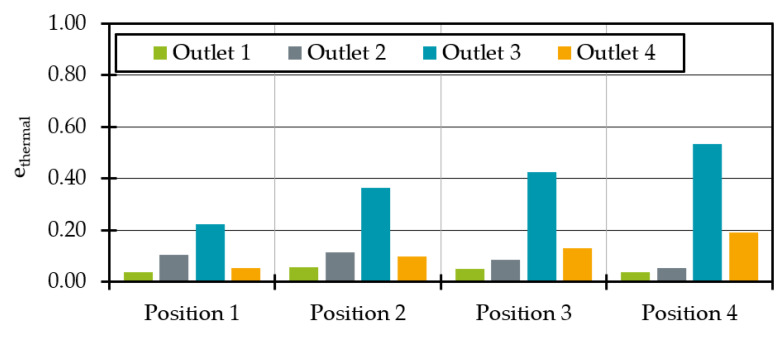
Thermal homogenisation indicator e_thermal_ at each outlet for different heat pipe positions.

The sum of the squared temperature differences of each cell to the mean temperature normalised to the cell area is calculated. In the case of a constant temperature in a cross section, σ = 0, in all other cases σ > 0. If e_thermal_ is negative, there is a less homogeneous temperature distribution at the outlet compared to the original temperature profile without heat pipe cooling. If no change in temperature homogeneity is calculated, e_thermal_ = 0. If, on the other hand, 0 < e_thermal_ < 1, the thermal homogeneity at the outlet with the heat pipe is better than without the heat pipe. In the special case e_thermal_ = 1, the melt has a perfectly homogeneous temperature at the outlet with the heat pipe. When comparing the thermal homogeneity (Figure 11) it is noticeable that the addition of a heat pipe improves the thermal homogeneity at all outlets regardless of the position. As expected, the influence is greatest for any heat pipe position at outlet 3. In agreement with the average temperatures, the homogeneity increases with the melt-carrying surface influenced by a heat pipe. This leads to an e_thermal_ of 0.53 for outlet 3 at heat pipe position 4. Moreover, the homogeneity for outlet 4 at position 4 is increasing. For outlet 1 and 2 the maximum homogeneity is reached at position 2.

**Figure 12:**
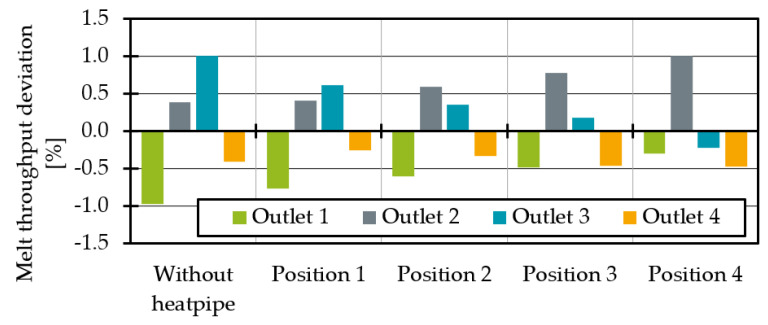
Melt throughput deviation at each outlet for different heat pipe positions.

Finally, the temperature changes will influence the melt throughput distribution. Therefore, the mass throughput deviations with dependence on the heat pipe positions are shown in Figure 12. For a perfectly homogenous distribution, the deviation should be 0% at all outlets. It is obvious that as the influence of the heat pipe on the melt channels increase, the throughput at outlet 3 decreases. This was to be expected since the temperature and thus the shear viscosity drop. Since the viscosity describes the flow resistance, throughput decreases at lower viscosities or higher flow resistances. However, the heat pipe influence at position 4 is so great that the system is overcompensating. The mass flow deviation at outlet 3 drops into the negative.

**Figure 13:**
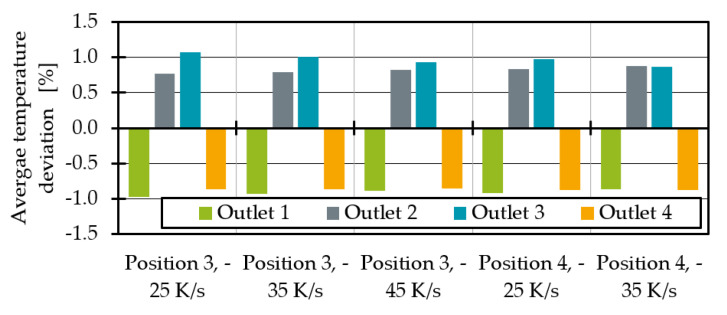
Average temperature deviation for different heat pipe performances and positions.

**Figure 14:**
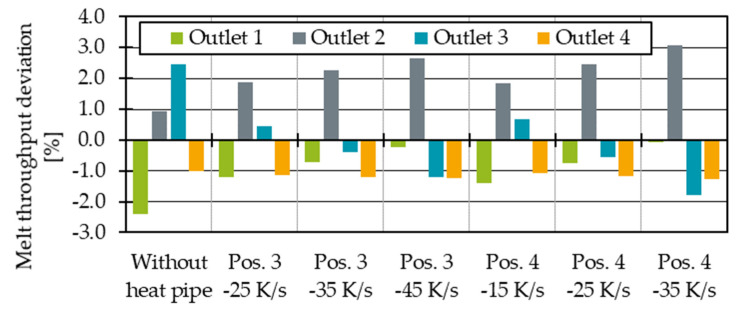
Melt throughput deviation for different heat pipe performances and positions.

All in all, the first simulations show that it is possible to influence the temperature and melt distribution with heat pipes. In terms of thermal homogeneity, positions 3 and 4 seem to be particularly promising. In the following, the heat transfer capacity of the heat pipes at position 3 and 4 is varied, starting with an increasing cooling rate at position 3 from −25 K/s to −35 K/s, which corresponds to the heat transfer capacity of 44.4 W and 62.1 W. The increasing heat transfer capacity has the effect of a further temperature drop at outlet 3 (Figure 13). At the same time, the temperature at outlet 2 rises. One explanation is a mass throughput redistribution (Figure 14). Due to the lower temperature at outlet 3, the flow resistance at that outlet increases, caused by higher viscous forces. Therefore, the output at outlet 2 increases, which is leading to an increasing viscous shear heating at that very same outlet. As shown in Figure 14, the increasing cooling rate leads to a linearly lower mass flow at outlet 3. At the same time, the melt is redistributed to outlets 1 and 2 where the melt flow also increases linearly. The mass flow at outlet 4 remains nearly unaffected. The parameters of the linearisation in Equation (14) are given in Table 1 and could be further investigated in the future to predict the required heat pipe performances to achieve optimal mass flow distributions for different process points.

(14) Melt throughput deviation=Cooling rate × x+y,

A similar, slightly weaker effect can be observed for the average temperatures (Figure 13). The temperature change dependent on the cooling rate can also be linearised (Equation (15)). The corresponding parameters are given in Table 2. Due to the small number of process points on which the linearisation is based, this correlation should be confirmed by further investigation.

(15) $$\Delta T_{\mathrm{average}\;}=\mathrm{Cooling}\;\mathrm{rate}\;\times \;x+y,$$

**Figure 15:**
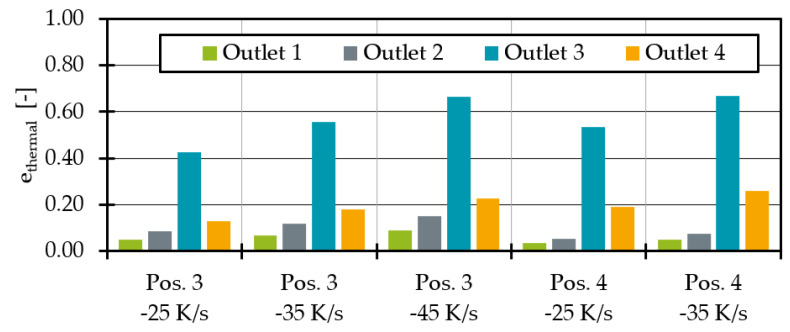
Thermal homogenisation indicator for different heat pipe performances and positions.

The analysis of the simulation results with a heat pipe at position 4 shows again the larger heat flux surface at this heat pipe position. The melt flows are increasing and decreasing more strongly, but the same effects tend to be observed. With a cooling rate of −35 K/s, it is possible to equalise the average temperatures in outlets 2 and 3. To achieve similar average temperatures at heat pipe position 3, the cooling rate was raised to −45 K/s (79.9 W). The remaining temperature difference between outlet 2 and 3 is 0.2 K. In addition to mass flow redistribution, the decreasing average temperatures at outlet 3 result in increasing thermal homogeneity (Figure 15). The heat pipes at position 3 seem to result in a more homogeneous temperature distribution at all outlets compared to position 4.

**Figure 16:**
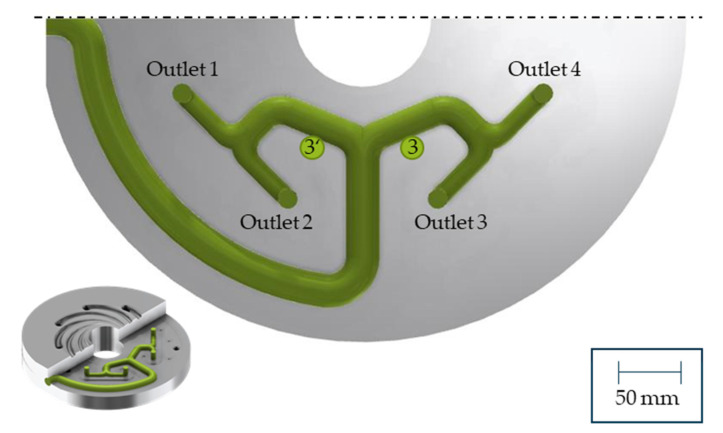
Heat pipe position for two heat pipes.

As the output at position 2 represents a new, excessively high mass flow, the integration of another heat pipe could be helpful to homogenise the temperature and mass flow distribution. Therefore, the integration of another heat pipe is tested by integrating two heat pipes symmetrically, as shown in Figure 16. To cool down the temperature at outlets 2 and 3, and since the average temperature at outlet 3 is higher than at outlet 2 (Figure 6), the cooling rates of the heat pipes at position 3′ are set lower than at position 3. The difference between the two positions is 10 K/s and 20 K/s, respectively. Maximum cooling rates of up to −70 K/s are simulated, with all cooling rates listed in Table 3. The extrusion operating point remains the same.

**Figure 17:**
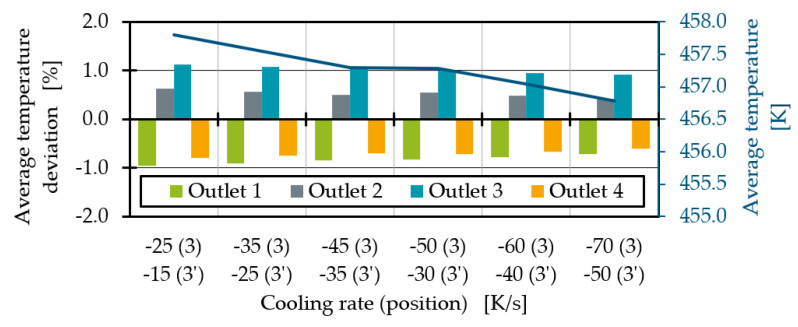
Average temperature deviation for two heat pipes with different performances at position 3 and 3′.

By integrating a second heat pipe, the temperature at outlet 2 decreases as expected (Figure 17). By increasing the cooling rates, the average temperatures converge further. A maximum cooling rate at which the average outlet temperatures fall below the optimum temperature is not yet reached in the process points investigated. For all selected cooling rates, the difference between the average temperature at outlet 2 and 3 remains between 0.8 K and 1 K. The temperature differences seem to tend to be slightly higher for the cooling rate difference of 20 K/s.

By increasing the cooling rate difference between position 3 and 3′, the average temperatures converge further. The average temperature deviation for the cooling rate combination of −60 K/s and −40 K/s is less than 1%.

**Figure 18:**
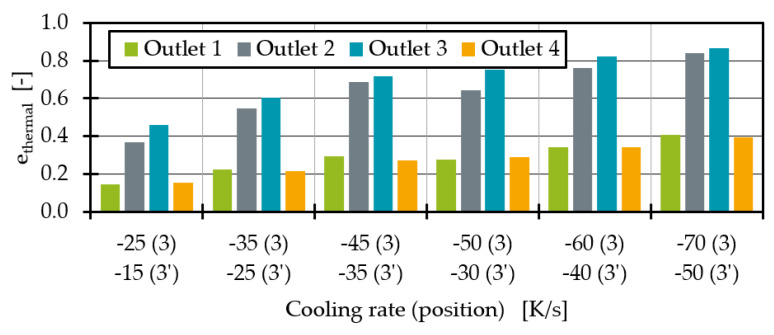
Thermal homogenisation indicator for two heat pipes with different performances at position 3 and 3′.

**Figure 19:**
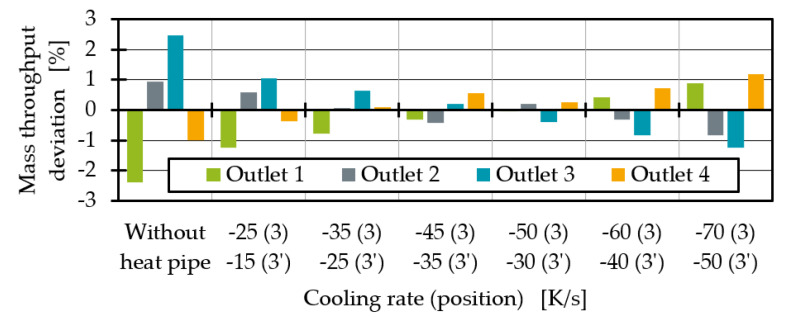
Average throughput deviation for two heat pipes with different performances at position 3 and 3′.

As shown in Figure 18, thermal homogenisation is improving for all outlets when increasing the cooling rates. Moreover, higher cooling rate differences between position 3 and 3′ seem to result in more homogenous temperatures at all outlets. The most homogenous process point is reached with a cooling rate of −70 K/s at position 3 and −50 K/s at position 3′. The mass flow deviation (Figure 19) shows that the mass flow distribution is significantly improved by the heat extraction of the two heat pipes at position 3 and 3′ compared to the state without heat pipes. The mass flow deviation for all test points with two heat pipes is less than 1%. Compared to one heat pipe (Figure 14), the mass flow distribution is no longer overcompensated and significantly improved. Especially at cooling rates of −45 K/s at position 3 and −35 K/s at position 3′, to then −50 K/s at position 3 and −30 K/s at position 3′, respectively, the mass flow distribution is excellent. At higher cooling rates, the throughput deviation becomes worse again for the process point investigated but is still acceptable compared to the distribution without heat pipes.

## 4. Conclusions

Inhomogeneous die temperatures lead to an inhomogeneous temperature distribution at pre-distributor outlets, and thus to an inhomogeneous distribution of mass flow over the pre-distributor outlets. This is caused by the externally applied heating and simultaneous flow of cold blowing air through the die centre as well as local shear heating of the melt. Since the temperature has a major influence on the polymer viscosity, it affects not only the flow resistances and thus the mass flow distribution, but also the extensional viscosity in the film bubble. During inflation, the melt parts of lower extensional viscosity can be stretched more easily, resulting in thin spots in the film. To avoid an inhomogeneous film thickness distribution and to achieve good film qualities, thermal homogenisation of the melt is necessary in the pre-distributor. Therefore, a new approach for cooling hot spots with heat pipes was investigated. Our simulations show that heat pipes can be used to influence melt temperatures at those locations where a temperature reduction is required. Two suitable heat pipe positions were identified that improve the homogeneity of the melt temperature by using only one heat pipe per die half. In addition to the heat pipe position, the heat pipe performance has a major influence on the temperature distribution and the melt flow distribution. With one heat pipe with a performance of 79.9 W the average temperature drops, but no thermal homogenization is achieved. Instead, the originally maximum flow at one of the outlets falls below the optimal average flow per outlet, and the melt flow deviation is overcompensated. Furthermore, the simulations show that both the average temperatures and the melt throughput deviation can be predicted with the cooling rates. Since the outlets of a pre-distribution die interact, one heat pipe is not sufficient to fully homogenise the temperature at each outlet to similar temperatures. Cooling the melt stream with the highest temperature creates a new maximum temperature and melt flow at another outlet. This changes as soon as a second heat pipe is integrated to cool the new maximum melt flow outlet. With heat pipe cooling of −60 K/s and −40 K/s, a maximum average temperature deviation and melt flow deviation of less than 1% can be achieved for all outlets. Whether our assumption that two heat pipes are sufficient for thermal homogenisation and also applies to other process points must be investigated in further simulation studies. For other process points, at least one heat pipe per outlet could be required in perspective in order to equalise the temperature at all outlets independently of the process point. Of course, the heat pipe performances would still have to be adjustable via cooling and set depending on the process point.

Due to the small number of process points investigated, the observed linear correlation between the cooling rate and the resulting average temperature and melt flow deviation should be confirmed by further CFD simulations, but also in practical tests. For future practical tests, further investigations are necessary to also develop a prediction of the average temperature and melt flow deviation for systems with more than one heat pipe per die half. For the practical tests, a 2^3^ pre-distributor will be retrofitted with heat pipes at the simulated suitable positions. An external air cooling system will be developed to cool the heat pipes. For this purpose, bent heat pipes will be used coming out of the side of the die where a pipe system with cool air flow will cool them. One challenge will be the isolation against die surfaces where cooling through the heat pipes is not desired. Another limitation is the available space around the pre-distributor in industrial blown film extrusion lines. In addition, the spiral die also has an influence on the temperature deviation. Another simulation setup with a spiral die could help to estimate the thermal influence in the downstream flow channel.

## Figures and Tables

**Table 1:** Linearisation parameters to predict the melt throughput deviation in case of one heat pipe at position 3.

Outlet	x	y	R^2^
1	0.49	−1.69	1
2	0.39	1.5	1
3	−0.82	1.25	1
4	−0.06	−1.06	0.9996

**Table 2:** Linearisation parameters to predict the average outlet temperatures in case of one heat pipe at position 3.

Outlet	x	y	R^2^
1	0.041	−1.02	1
2	0.023	0.75	0.9999
3	−0.070	1.14	1
4	0.005	−0.87	0.9998

**Table 3:** Heat pipe performances and process point for simulations with two heat pipes.

Material	Throughput	Melt Temperature	Die Temperature	Support Air Temperature	Cooling Rate Position 3	Cooling Rate Position 3′
LDPE 2101N0W	100 kg/h	453.15 K	473.15 K	298.15 K	−25 K/s	−15 K/s
					−35 K/s	−25 K/s
					−45 K/s	−35 K/s
					−50 K/s	−30 K/s
					−60 K/s	−40 K/s
					−70 K/s	−50 K/s

## Data Availability

The data that support the findings of this study are available from the corresponding author, L.L., upon reasonable request.
